# Specialty preferences among medical students in a Kenyan university

**Published:** 2010-06-08

**Authors:** Philip Maseghe Mwachaka, Eric Thuo Mbugua

**Affiliations:** 1 School of Medicine, University of Nairobi, Kenya

**Keywords:** Specialty, Gender, Career, Medical study, Kenya

## Abstract

**Background:**

Specialty distribution in Kenya continues to exhibit gender disparities despite the increasing number of female medical students graduating each year. This study aimed at assessing specialty preferences and factors influencing these choices among male and female medical students in Kenya.

**Methods:**

Four hundred and fifty medical students, from first to fifth year of study at the University of Nairobi, were each issued a self-administered questionnaire designed to assess their specialty preferences and factors influencing these choices. The specialty preferences were compared with the actual distribution of specialists in Kenya. Data collected were analyzed using Statistical Package for Social Sciences.

**Results:**

Three hundred and eighty five (85.6%) questionnaires were completed. Surgery had the highest preference rate followed by pediatrics, internal medicine and obstetrics and gynecology. Significantly more males preferred surgery than females who mainly selected pediatrics (p<0.001). There was an increased likelihood of female students choosing controllable lifestyle specialties. These preferences mirrored the actual distribution of specialists in Kenya. Male students significantly considered prestige in a specialty (p=0.006), while their female counterparts mostly considered ease of raising a family and gender distribution in the specialty (p<0.001).

**Conclusion:**

Gender-based similarities and differences exist in factors influencing specialty preferences among Kenyan medical students. These factors may explain the observed specialist doctor distribution in the country.

## Background

Developing countries especially in Sub Saharan Africa are faced with a critical shortage of skilled health personnel. Kenya is ranked as one of the countries with poorest doctor to patient ratio ranging between 1:25,000 in Nairobi (capital city) and 1:308,878 in rural areas [1,2]. A survey done showed that specialized medical personnel, such as physicians, surgeons, obstetric gynecologists, pediatricians, anesthetists and ophthalmologists, represent a mere 5% of total health workforce [2]. This clearly depicts a deficiency of medical specialists in Kenya.

One of the measures taken by the government to address this shortage was to train more medical personnel. According to an economic survey by the Kenya Bureau of Statistics 2007, the total number of medical students training to be doctors in 2000 was 1355 and increased to 2,098 in 2006 [2,3]. In this survey, there was a noted increase in the number female students training, from 488 in 2000 to 984 in 2006. In spite of this drastic increase in number of female medical students, specialist distribution in the country continues to exhibit gender disparities [4].

In order to correct the maldistribution of doctors by specialty, factors influencing choice and preference of specialty by doctors and medical students should be identified [5]. Although several studies have been done to assess the factors that influence career choices among medical students [5-9] there is scarcity of data from countries in Sub Saharan Africa. These studies are based on developed countries whose health care demands differ from developing countries. This study aimed at determining specialty preferences and factors influencing these choices among medical students in Kenya.

## Methods

Setting and participants: University of Nairobi is the largest and oldest university in Kenya. Its College of Health Sciences based at Kenyatta National Teaching and Referral Hospital offers a five year undergraduate degree in medicine and surgery. Students in this program chose of medicine in their final year of high school or immediately after high school. With the increasing demand for doctors, the number of students admitted to this program has also been increasing. In the 2008/09 academic year, there were 1557(874 male and 683 female) students. For this study we enrolled 450 students, ninety per year of study. The survey was conducted between September and October 2009, coinciding with the last quarter of the 2008/09 academic year. All participants were informed of the aim of the study and that their involvement was voluntary. This survey did not require ethical approval because the data collection was anonymous.

Procedure and Measures: Self administered questionnaires (printed) were disseminated and collected in classrooms for 1^st^ to 5^th^ year students. The survey took approximately 10 minutes to complete. Information collected included: gender, marital status, year of study, the specialty they are interested in, factors that influenced this choice, and timing of specialty choice. The participants were offered the following list of possible specialties: surgery, internal medicine, pediatrics, obstetrics and gynecology, public health, psychiatry, radiology, anesthesiology, pathology, microbiology, anatomy, physiology, biochemistry, ophthalmology, immunology and other (a write in option). Option for ‘not yet decided’ was also included. These specialties were preselected as it was felt they would be clear and distinct for most students. The participants were allowed to choose only one specialty.

Regarding the factors influencing choice of the specialties, the students responded to the question “Did this factor influence your choice of the specialty?” The response was either “yes” or “no” to a list that included: encouragement by teaching or clinical staff, role model in the specialty, job opportunities and financial rewards, prestige of the specialty, academic and research opportunities, intellectual challenge in the specialty, lifestyle of practice, gender distribution in the specialty, ease of raising up a family, ease of entry into residency, length of residency, lifestyle during residency, and further training required after residency. These factors were based on similar published studies [5-9].

Analysis: Data collected were analyzed using Statistical Package for Social Sciences version 17.0. We used the modified Schwartz et al [9] method to classify the specialties as having either a controllable or uncontrollable lifestyle. Specialties with uncontrollable lifestyles included surgery, pediatrics, internal medicine, and obstetrics and gynecology. The remaining medical specialties were grouped into controllable lifestyle careers. Chi square test was used to evaluate gender differences as well as compare between factors influencing choice of controllable and uncontrollable lifestyle careers. A p-value≤0.05 was considered statistically significant.

## Results

### Study demographics

Of the 450 questionnaires administered, 385 (85.6%) were completed and returned. Male respondents were 217 (56.4%) while females were 168 (43.6%). The response according to year of study is summarized in Table 1. Sixteen (0.042%) students were married while the rest were single.

### Specialty preferences of medical students

Table 2 illustrates the specialty preferences among the students as well as the actual distribution of specialists in Kenya. Most students, 319(82.8%), preferred a medical specialty while 8(2.1%) selected non medical careers. The non medical specialties chosen were business (5 students), law (2 students) and music (1 student). Fifty eight (15.1%) students were undecided on their future specialties. Specialties more popular among male students were surgery 76(40.6%) and internal medicine 29(15.5%). Female students were more interested in pediatrics 40(28.6%) followed by surgery 29(20.7%).

Significant gender differences were observed in the choice of surgery and pediatrics (p<0.001). Male students had a double-fold likelihood of choosing surgery, while female students had a five-fold chance of choosing pediatrics. There was also an increased likelihood of female students preferring controllable lifestyle specialties such as ophthalmology (odds ratio 3.13), pathology (odds ratio 2.70), radiology (odds ratio 1.82), microbiology (odds ratio 1.41) and physiology (odds ratio 1.41). These specialty preferences among male and female students mirrored the actual gender distribution of specialists in Kenya (Table 2).

### Factors influencing specialty preferences

Specialty preferences among both male and female students was mainly influenced by presence of a role model in specialty, job opportunities and financial rewards, intellectual challenge in the specialty, and research opportunities in the specialty of choice (Table 3). In addition, compared to female students, male students selected specialties mainly because of prestige of specialty (p=0.006). Female students on the other hand mostly considered ease of raising a family (p<0.001), and gender distribution in the specialty (p<0.001).

Table 3 also illustrates the factors influencing choice of controllable and uncontrollable lifestyle specialties. Female students significantly preferred controllable lifestyle careers than males because of ease of raising a family (p*<0.001*) and gender distribution in these specialties (p=0.023). Preference of uncontrollable lifestyle among male students was largely due to prestige of the specialty (p=*0.006*), while among female students was mainly due to length of residency (p=*0.010*) and gender distribution in the specialty (p*<0.001*).

**Table 1:** Response rate according to the year of study

**Year of study**	**Male n (%)**	**Female n (%)**	**Total n (%)**
First	38 (55.9%)	30 (44.1%)	68 (100%)
Second	52 (58.4%)	37 (41.6%)	89 (100%)
Third	39 (56.5%)	30 (43.5%)	69 (100%)
Fourth	55 (65.5%)	29 (34.5%)	84 (100%)
Fifth	33 (44.0%)	42 (56.0%)	75 (100%)
**Total**	**217 (56.4%)**	**168 (43.6%)**	**385 (100%)**

**Table 2:** Specialty preferences among students and specialist distribution in Kenya

	**Student specialty preferences**				**Actual practice in the country**			
Specialty	**Male n (%)**	**Female n (%)**	**Total n (%)**	***p-value***	**Male n (%)**	**Female n (%)**	**Total n (%)**
Surgery	76 (35.0)	29 (17.3)	105 (27.3)	*<0.001*	386 (30.3)	11 (3.9)	397 (25.5)
Pediatrics	10 (4.6)	40 (23.8)	50 (13.0)	*<0.001*	129 (10.1)	84 (29.7)	213 (13.7)
Internal medicine	30 (13.9)	17 (10.1)	47 (12.2)	*0.33*	183 (14.4)	44 (15.5)	227 (14.6)
Obstetrics & Gynecology	28 (12.9)	17 (10.1)	45 (11.7)	*0.399*	254 (19.9)	49 (17.3)	303 (19.5)
Public Health	13 (6.0)	6 (3.6)	19 (4.9)	*0.277*	18 (1.4)	6 (2.1)	24 (1.5)
Pathology	5 (2.3)	9 (5.4)	14 (3.6)	*0.102*	35 (2.7)	12 (4.2)	47 (3.0)
Ophthalmology	3 (1.4)	7 (4.2)	10 (2.6)	*0.089*	57 (4.5)	19 (6.7)	76 (4.9)
Microbiology	4 (1.8)	3 (1.8)	7 (1.8)	*0.967*	5 (0.4)	1 (0.4)	6 (0.4)
Radiology	3 (1.4)	4 (2.4)	7 (1.8)	*0.467*	56 (4.4)	18 (6.4)	74 (4.8)
Psychiatry	5 (2.3)	1 (0.6)	6 (1.6)	*0.179*	49 (3.8)	14 (4.9)	63 (4.0)
Anesthesiology	2 (0.9)	2 (1.2)	4 (1.0)	*0.796*	84 (6.6)	21 (7.4)	105 (6.7)
Dermatology	0 (0.0)	1 (0.6)	1 (0.3)	*0.255*	18 (1.4)	4 (1.4)	22 (1.4)
Medical research	3 (1.4)	1 (0.6)	4 (1.0)	*0.45*	-	-	
Non medical	5 (2.3)	3 (1.8)	8 (2.1)	*0.724*	-	-	
Not yet decided	30 (13.8)	28 (16.7)	58 (15.1)	*0.439*	-	-	
**TOTAL**	**217 (100)**	**168 (43.6)**	**385 (100)**		**1274 (100)**	**283 (100)**	**1557 (100)**

**Table 3:** Factors influencing specialty preferences

	**All specialties**			**Uncontrollable lifestyle**			**Controllable lifestyle**		
Specialty	**Male n (%)**	**Female n (%)**	***p-value***	**Male n (%)**	**Female n (%)**	***p-value***	**Male n (%)**	**Female n (%)**	***p-value***
Encouragement by staff	64 (45.7)	76 (40.6)	*0.359*	47 (45.6)	63 (44.1)	*0.806*	17 (50.0)	12 (30.8)	*0.128*
Role model in the specialty	92 (65.7)	128 (68.4)	*0.554*	75 (72.8)	104 (73.2)	*1.000*	16 (47.1)	22 (56.4)	*0.441*
Job and financial rewards	84 (60.0)	128 (68.4)	*0.114*	61 (59.2)	99 (69.2)	*0.104*	20 (58.8)	26 (66.7)	*0.726*
Prestige of the specialty	57 (40.7)	105 (56.1)	0.006*	46 (44.7)	89 (62.2)	0.006*	9 (26.5)	12 (30.8)	*0.528*
Lifestyle of practice	98 (70.0)	109 (58.3)	0.030*	68 (66.0)	80 (55.9)	*0.111*	27 (79.4)	26 (66.7)	*0.126*
Ease of raising a family	86 (61.4)	73 (39.0)	<0.001*	26 (25.2)	39 (27.3)	*0.722*	29 (85.3)	17 (43.6)	<0.001*
Intellectual challenge	100 (71.4)	121 (64.7)	*0.199*	76 (73.8)	97 (67.8)	*0.313*	21 (61.8)	20 (51.3)	*0.346*
Length of residency training	57 (40.7)	57 (30.5)	*0.055*	41 (39.8)	35 (24.5)	0.010*	15 (44.1)	20 (51.3)	*0.544*
Ease of entry into residency	26 (18.6)	30 (16.0)	*0.550*	16 (15.5)	17 (11.9)	*0.408*	9 (26.5)	12 (30.8)	*0.802*
Lifestyle during residency	40 (28.6)	57 (30.5)	*0.709*	26 (25.2)	39 (27.3)	*0.722*	12 (35.3)	17 (43.6)	*0.778*
Further training after residency	54 (38.6)	78 (41.7)	*0.568*	61 (39.8)	64 (44.8)	*0.439*	11 (32.4)	14 (35.9)	*0.725*
Gender distribution in specialty	57 (40.7)	21 (11.2)	<0.001*	44 (42.7)	15 (10.5)	<0.001*	11 (32.4)	6 (15.4)	0.023*
Academic or research opportunities	87 (62.1)	125 (66.8)	*0.380*	65 (63.1)	96 (67.1)	*0.512*	20 (58.8)	29 (74.4)	*0.549*

* Statistically significant.

## Discussion

Choosing a career is a complex process and may be influenced by several factors. This study aimed at determining specialty preferences and factors influencing these choices among male and female medical students in Kenya. We found significant gender differences in the choice of surgery and pediatrics. Male students had a special liking for surgery while their female counterparts preferred pediatrics. Previous studies in other countries have also reported similar gender differences in doctors’ and medical students’ specialty choices and preferences [5-8,10]. Some factors such as control of lifestyle and work balance have been identified as been related to women’s specialty preferences and choices [9-11].

Even though in our study most students, both male and female, preferred uncontrollable lifestyle specialties, there was a higher likelihood female students selecting a controllable lifestyle specialty. Controllable lifestyle careers have been defined as those that allow more personal time free of practice requirements for leisure, family, and control of total weekly hours spent on professional responsibilities [7,9]. Studies have further shown that women are more likely to integrate family responsibilities with a career, and therefore they consider flexibility of work and opportunity for part time working in their choice of careers [11-14]. In agreement with these authors, female students in the current study significantly considered specialty’s lifestyle of practice and easy of raising a family.

Role models especially of the same gender have been reported as a key factor in career choice [11,12]. Female students are discouraged from specialties such as surgery as there are few female surgeons to look up to as role models [15,16]. Consequently these students turn to other specialties that have more female representation such as pediatrics [17]. This explains the observed high preference of pediatrics among female students in the current study. These students significantly considered specialty’s gender distribution. Studies have also shown that women suffer more gender discrimination than males in male dominated specialties, and this has been reported to deter their choice of these specialties [18,19].

Even though pediatrics was the most popular area amongst female students, surgery was the second most popular with up to two fifths of the female students selecting it. The popularly held notion that surgery is ‘men only club’ [15,16] may become history if more females take up surgical careers. It is possible that with the increasing number of female medical graduates some venture in previously male dominated areas and provide role models for those still training. Factors driving women to previously male dominated specialties appear to be similar to the ones driving men to the same specialties. These include job opportunities and financial gains, intellectual challenge, and availability of academic and research positions.

The results of this study should be viewed in the context of the following limitations. Firstly we measured specialty preference at one point in time. It is known from literature that specialty choice does not remain stable over the course of medical education. Students tend to use their clinical years as well as internship period to refine their specialty preferences. Secondly, the study was only conducted in one medical school. Thus, the results may not be generalized to the entire country. However this study serves as a pilot for future, more comprehensive cohort studies following up the students from the early years in medical school to the actual time they choose the specialties.

We also dichotomized selected specialties into controllable or uncontrollable lifestyle. This classification is an oversimplification as lifestyle among and within the specialties is variable. Other ways of classifying specialties have been introduced such as technique oriented versus person oriented, and primary care versus non primary care specialties [20,21]. However, these classifications also have their own limitations. Even though classifying specialties according to lifestyle is subjective, multiple studies have validated this designation used by Schwartz and colleagues [5,7,9,11,12,22].

## Conclusion

Our study has revealed similarities and differences in specialty preferences and factors influencing these choices among male and female students in Kenya. Specialty preference among the students is corresponds to the specialist doctor distribution in Kenya. Enthusiasm and encouragement from a role model has the ability to give students the necessary conviction that they can succeed in any discipline. Thus, barriers caused by a lack of same sex role models in certain specialties must be recognized and addressed. There is also need for more career education in order to match the career preferences of students with the demands of the labor market.

## Competing interests

The authors declared they have no conflicts of interests.

## Authors’ contributions

PM: Literature review, Data collection, Data analysis, Manuscript write up, EM: Research question, Data collection, Data analysis, Manuscript write up.

## Acknowledgments

The authors would like to acknowledge all medical students at the University of Nairobi-Kenya who took part in the study.
